# Urethral Calculi

**DOI:** 10.5811/cpcem.2019.5.43182

**Published:** 2020-02-24

**Authors:** Perry Lee, Jordana Haber

**Affiliations:** University of Las Vegas, Department Emergency Medicine, Las Vegas, Nevada

## Abstract

Urolithiasis is a condition with calculi commonly found within the kidney, ureter, or bladder. The urethra is an uncommon location of urolithiasis, with limited case reports and literature reviews of its presentation and management. Here we discuss a 24-year-old female who presented with urinary urgency, flank pain, and urinary retention for 12 hours. Physical exam showed a calculus at the urethral meatus. This case discusses the manual removal of a urethral calculus in a female patient with use of forceps, resulting in complete resolution of symptoms and urinary retention.

## INTRODUCTION

Urolithiasis is a common condition seen in the emergency department (ED) that most often presents with calculi located in the kidney, ureter, or bladder. An obstructing calculi located at the urethra is an uncommon presentation where the management options are unique to its location.^1^ There are limited case reports and literature reviews of urethral calculi and management in the emergency setting. In this case report, we discuss the presentation, physical exam, imaging studies, diagnosis, and manual removal of a urethral calculi.

## CASE REPORT

A 24-year-old female with past medical history of nephrolithiasis presented to the ED complaining of urinary urgency, left flank pain, and urinary retention over the previous 12 hours. She spontaneously passed a renal stone one week prior at home with planned follow-up with her urologist in three days. She had been taking nitrofurantoin over the past year as prophylaxis for recurrent urinary tract infections.

Initial vital signs included temperature of 37.1^o^ Celsius (98.7^o^ Ferenheit), heart rate 104 beats per minute, blood pressure 126/87 millimeters of mercury, and respiratory rate 22 breaths per minute. Initial physical exam showed a mildly uncomfortable appearing female who was non-toxic. She had left flank tenderness to deep palpation with no midline bony spinal tenderness. Abdomen was soft, non-tender, and non-distended with no palpable pulsatile mass.

**Image 1:**
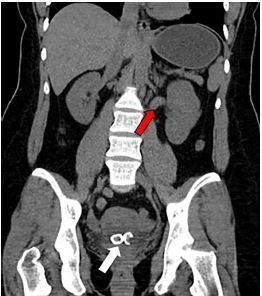
Computed tomography of the abdomen and pelvis without contrast (coronal view) showing bladder calculi (white arrow) and hydroureter (red arrow).

The patient’s blood work ordered by the triage physician was significant for a leukocytosis with white blood cell count of 14.3 thousand per cubic millimeter (K/mm^3^) (reference range 3.7 to 10.6 K/mm3). All other blood work was within normal limits, including a creatinine of 0.8 milligrams per deciliter (mg/dL) (reference range 0.6 to 1.5 mg/dL). Pregnancy test was negative. Computed tomography (CT) of the abdomen and pelvis without contrast ordered by the triage physician showed a distended urinary bladder with multiple bladder stones and mild left hydroureter (Image 1).

**Image 2:**
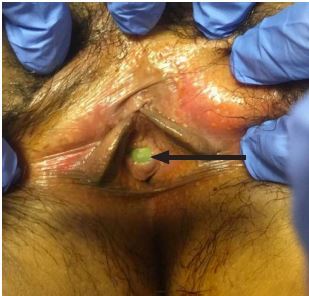
Urethral calculus at the urinary meatus on external pelvic exam (arrow).

No urine sample had been provided for a urine analysis after two hours of arrival and a catheter sample was ordered. The patient’s nurse reported difficulty finding the urethra for catheter placement and requested assistance. A pelvic exam was then performed showing a calculus at the urethral meatus (Image 2).

**Image 3:**
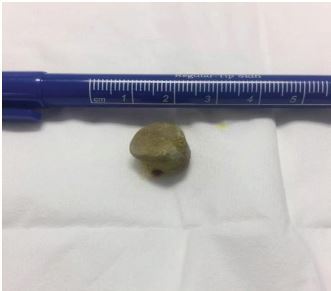
15-millimeter urethral calculus post-manual removal.

An initial attempt to manually remove the calculus using topical lidocaine gel and direct pressure on the stone through the vaginal canal was unsuccessful. A second attempt at calculus removal was successful by grasping the calculus with a hemostatic forceps (Image 3).

The patient was able to immediately void without difficulty after the 15 mm calculus was removed. She was discharged in improved condition with an outpatient urology follow-up.

## DISCUSSION

Urolithiasis is a common condition with an increasing number of ED visits reported over the past decade.^2^ It is well established that stone size and location are the major determinant of spontaneous passage with most calculi less than 5 mm passing spontaneously and calculi more than 10 mm unlikely to spontaneously pass.^3^,^4^ Additionally, there has been an increase in prevalence of women diagnosed with urolithiasis in a previously male-predominant disease.^5^

Urethral calculi are the rarest presentation of urolithiasis with no established prevalence and no standardized management and treatment.^1^ In a literature review, we found the presentation and management of urethral calculi to be limited to case reports and small literature reviews. Additionally, the majority of case reports are of male patients. One case report documented a 22-year-old male presenting with penile pain and urinary retention, who was found to have a urethral calculus on bedside ultrasound and ultimately underwent lithotripsy by urology.^5^ Another reported a 54-year-old male with a urethral calculus who underwent open surgery by urology for calculus removal.^7^ A final case report documented a 64-year-old female with urinary retention and a urethral calculus that could not be extracted manually. She required pneumatic lithotripsy through a rigid cystoscope under spinal anesthesia.^8^

A study of 34 male patients with urethral stones showed a safe and effective treatment approach by retrograde manipulation of the calculi with a 16 French foley urethral catheter prior to endoscopic or extracorporeal shock wave lithotripsy.^9^ This approach requires general anesthesia and does carry intrinsic risk of bladder and urethral trauma.

**CPC-EM Capsule**

What do we already know about this clinical entity?

Urethral calculi are an uncommon presentation of a urolithiasis, a common condition that most often presents with calculi located in the kidney, ureter, or bladder.

What makes this presentation of disease reportable?

An obstructing calculi located at the urethra is an uncommon presentation where the management options are unique to its location.

What is the major learning point?

In specific cases, direct manual removal of a urethral calculus with forceps can be a definitive treatment while avoiding invasive treatment and possible associated complications.

How might this improve emergency medicine practice?

This case discusses a possible solution in an uncommon presentation with limited case reports and literature reviews of urethral calculi and management in the emergency setting.

## CONCLUSION

Urethral calculus is a rare presentation of urolithiasis. This rare case demonstrates successful management of a urethral calculus in a female patient by manual removal of the calculus with forceps. In specific cases, direct manual removal of a urethral calculus with forceps can be a definitive treatment resulting in complete resolution of symptoms and urinary retention while avoiding invasive procedures and possible associated complications.
